# Liraglutide-induced structural modulation of the gut microbiota in patients with type 2 diabetes mellitus

**DOI:** 10.7717/peerj.11128

**Published:** 2021-04-01

**Authors:** Junjie Shang, Fang Liu, Bing Zhang, Kunlun Dong, Man Lu, Rongfeng Jiang, Yue Xu, Le Diao, Jiangman Zhao, Hui Tang

**Affiliations:** 1 Nanyang Second General Hospital, Nanyang, Henan Province, China; 2 Kaifeng Central Hospital, Kaifeng, Henan Province, China; 3 Shanghai Biotecan Pharmaceuticals Co., Ltd., Shanghai, China; 4 Shanghai Zhangjiang Institute of Medical Innovation, Shanghai, China

**Keywords:** Type 2 diabetes mellitus, Liraglutide, Gut microbiota, 16S rRNA gene sequencing, HbA1c

## Abstract

Accumulating evidence has suggested the importance of gut microbiota in the development of type 2 diabetes mellitus (T2DM). In the present study, 40 patients with T2DM were treated with liraglutide for 4 months. Feces samples and clinical characteristics were collected from these 40 T2DM patients before and after the liraglutide treatment. The diversity and composition of gut microbiota in the two groups were determined by sequencing the V4 region of bacterial 16S rRNA genes. Meanwhile, blood glucose, insulin, hemoglobin A1c (HbA1c), and lipid metabolism were also measured in the pre- and post-liraglutide-treatment groups. We find that Baseline HbA1c was associated with liraglutide treatment response (*R*_2_ = 0.527, *β* =  − 0.726, *p* < 0.0001). After adjusted for baseline HbA1c, blood urea nitrogen was associated with liraglutide treatment response. Besides, our results showed reduced gut microbial alpha diversity, different community structure distribution and altered microbial interaction network in patients treated with liraglutide. The liner discriminant analysis (LDA) effect size (LEfSe) analysis showed that 21 species of bacteria were abundant in the pre-liraglutide-treatment group and 15 species were abundant in the post-liraglutide-treatment group. In addition, we also find that *Megamonas* were significantly correlated with older age, diabetes duration and diabetic retinopathy, *Clostridum* were significantly correlated with family history of diabetes and *Oscillospira* were significantly correlated with both diabetic retinopathy and diabetic peripheral neuropathy. Functional analysis based on Kyoto Encyclopedia of Genes and Genomes (KEGG) and cluster of orthologous groups (COG) annotations enriched three KEGG metabolic pathways and six functional COG categories in the post-liraglutide-treatment group. In conclusion, our research suggests that baseline HbA1c, blood urea nitrogen and gut microbiota are associated with the liraglutide treatment applied on patients with T2DM. These findings may contribute to the beneficial effects of liraglutide against diabetes.

## Introduction

Type 2 diabetes mellitus (T2DM) is a highly prevalent metabolic disorder characterized by the imbalance in blood glucose level and altered lipid profile (Sharma & Tripathi, 2019), which is caused by either disturbed insulin secretion, disturbed insulin effect, or usually both (Petersmann et al., 2019). The World Health Organization (WHO) has proposed that T2DM could be one of the top ten potent reasons of death worldwide by 2030 (Kelly et al., 2008). The increased morbidity and mortality rates related to T2DM are often associated with vascular complications, such as cardiovascular diseases (Zheng et al., 2018), nephropathy (Lu et al., 2017) and retinopathy (Maugeri et al., 2018). Approximately 50% of T2DM patients developed a diabetic peripheral neuropathy (DPN), which is a chronic and progressive disorder affecting the peripheral nervous system (Edwards et al., 2008). Diabetic retinopathy (DR) is a microvascular complication of diabetes mellitus and can cause blindness or visual impairment (Kulkarni et al., 2019). T2DM is thought to arise due to the overlaps of genetic factors, sedentary lifestyle, poor diet, excessive visceral obesity, and other environmental exposures throughout life (Salamon et al., 2018).

In recent years, gut microbiota has been found to play a critical role in the establishment and maintenance of human health. A large number of studies showed that the alteration of microbiota is associated with many human diseases including gastrointestinal diseases (Tong et al., 2013), cancer (Garrett, 2015), metabolic disease (Musso, Gambino & Cassader, 2011), neurodegenerative disorders (Hsiao et al., 2013) and cardiovascular (Wang et al., 2011). Previous studies of clinical T2DM cases have found compositional changes between the gut microbiome in patients and healthy controls (Karlsson et al., 2013). However, the pathophysiological mechanisms that link the microbiota to T2DM have not been well elucidated.

Liraglutide, a glucagon-like peptide 1 (GLP-1) analogue, was approved by the U.S. Food and Drug Administration in 2010 for treating T2DM (Vilsboll & Garber, 2012). GLP-1 is an incretin hormone which is secreted by intestinal L cells in response to food ingestion and it shows a significant advance in the treatment of T2DM (Drucker & Nauck, 2006). There is evidence that GLP-1 may play a role in the biological function of intestinal epithelium, correlating these effects with changes in gut microbiota (Hwang et al., 2015).

However, the effect of liraglutide on gut microbiota in T2DM is not fully understood. Therefore, we performed a randomized study in 40 individuals diagnosed with T2DM, and combined 16S rRNA gene amplicon sequencing to investigate the effects of liraglutide on the composition and function of the gut microbiota.

## Materials and Methods

### Study population

The 40 (including both male and female) T2DM patients were aged 25–83 years, with mean % HemoglobinA1c (HbA1c) concentration of 9.17% (76.67 mmol/mol) (5.3%) (34.43 mmol/mol) − 12.2% (109.84 mmol/mol), and body-mass index (BMI) scores >25 kg/m^2^. HbA1c is the gold standard of glycemic control index and the standardization of HbA1c was considered to be important, which was proceeded by the National Glycohemoglobin Standardization Program (NGSP) and the International Federation of Clinical Chemistry and Laboratory Medicine (IFCC) (Weykamp, 2013). All participants had been treated with metformin as a monotherapy (stable doses for 2 months). Relevant exclusion criteria included: taking other glucose-lowering medications and those who had been taking antibiotics in the last 3 months. The recruited participants were randomly assigned to receive 1.2 mg of liraglutide for 4 months. BaselineHbA1c was assessed at before treated-with liraglutide. HbA1c change (liraglutide treatment response) was calculated based on treatment HbA1c value (after 4 months treated-with liraglutide) minus baseline HbA1c value. All 40 T2DM patients were switched from oral metformin to subcutaneous injections of liraglutide for 4 months once daily. The metformin-liraglutide substitution design was applied because a pre-study feasibility assessment reported that it was impossible to recruit sufficient subjects with liraglutide as their primary medication for T2DM (Hodge et al., 2016). All procedures were performed in accordance with the ethical standards of the Clinical Research Ethics Committee of the Nanyang Second General Hospital, and written informed consents were obtained from all participants included in the study.

### Fecal DNA extraction and 16s rRNA gene sequencing

Total 80 fresh feces samples were collected in sterile collection tubes (Fisher Scientific, Waltham, MA, USA) before and after the 4-month liraglutide treatment and then stored at −20 °C until being transported to the research center in chilled styrofoam containers. They were subsequently stored on-site at −80 °C for further analysis. Microbial DNA was extracted from 200 mg fecal sample using the QIAamp PowerFecal Pro DNA Kit (QIAGEN) which contains a bead-beating step. Briefly, adding 200 mg fecal sample and 800 µl lysis buffer in a beads-containing tube and vortex at maximum speed for 10 min. Take 350 µL supernatant after 1 min centrifugation at 15,000 g and use it in the subsequent steps according to the kit instructions. DNA is finally eluted in 100 µl elution buffer for downstream applications. DNA was amplified using primers targeting the V4 region of the 16S rRNA gene (515F 5′-GTGYCAGCMGCCGCGGTA-3′, 806R 5′-GGACTACNVGGGTWTCTAAT-3′). PCR was run in a Veriti™ 96-Well Thermal Cycler PCR system (Thermo Fisher Scientific) using the following program: 95 °C for 3min, followed by 21 cycles of 95 °C for 30 s, 56 °C for 30 s, 72 °C for 30 s, with a final extension at 72 °C for 5 min. Mixed amplicons were pooled and the sequencing was conducted at Shanghai Biotecan Pharmaceuticals Co., Ltd. (Shanghai, China) using an Illumina Novaseq 6000 Sequencing system (Illumina, USA) according to the manufacturer’s instructions.

### Sequence data analysis

Sequences were assigned to operational taxonomic units (OTUs) with 97% similarity (Greengenes database: http://greengenes.lbl.gov) into mothur (v.1.39.5), OTU taxonomy was assigned to the Greengenes database for the comparisons, using the Quantitative Insights into Microbial Ecology (QIIME) software package (Caporaso et al., 2010). Both Kyoto Encyclopedia of Genes and Genomes (KEGG) (Langille et al., 2013) and Clusters of Orthologous Groups of proteins (COG) (Liu et al., 2019) pathways were categorized using Phylogenetic Investigation of Communities by Reconstruction of Unobserved States (PiCRUSt) and both were imported into STAMP (v.2.1.3) for visualization. To identify taxa with differing relative abundances between the two groups, linear discriminant analysis (LDA) effect size (LEfSe) analyses were performed on the website (http://huttenhower.sph.harvard.edu/galaxy). The cut off value was the absolute LDA score (log10) >3.0 with a *p* < 0.05. The alpha diversity “summary.single” script was used to calculate ACE, Chao1, Shannon and Simpson indexs in the mothur software package. “Beta_diversity.py” script was used to calculate the beta diversity in the QIIME software, and the Principal Coordinate Analysis (PCoA) was used to measure beta diversity in each group. Coabundance network analysis was based on Spearman correlation. Only genera present in at least 60% of samples were used for correlation analysis, and only connections with a *r* > 0.6 or < −0.6 and *P* < 0.05 were used for network building on the basis of Igraph R package and imported into Cytoscape (v.3.6.0) for visualization.

### Statistical analysis

The data are shown as the mean ± standard error of the mean (SEM). Bacterial taxa that were differentially abundant were identified by using the Wilcoxon rank-sum test (for two groups) or Kruskal-Wallis test (for more than two groups) in R studio (v.3.6.1). Clinical data analyses between the two groups were conducted by Wilcoxon signed–rank test or *t*-test using Prism version 6.0 (GraphPad, San Diego, CA, USA). Linear regression analysis was performed using R studio (v.3.6.1). A *p* < 0.05 was considered statistically significant.

## Results

### Baseline HbA1c is a key predictor of liraglutide treatment response

**Figure 1:**
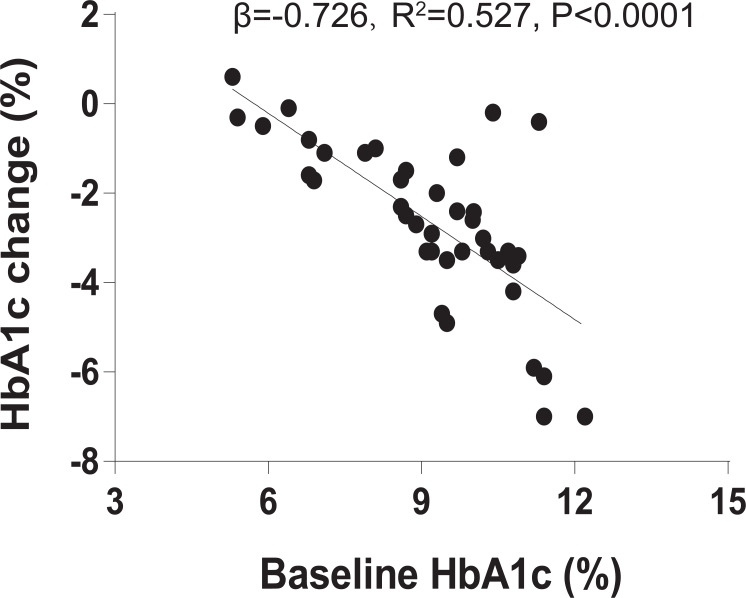
Baseline HbA1c is associated with liraglutide treatment response. The association between % baseline HbA1c and % HbA1c change using linear regression analysis (*R*^2^ = 0.527, *β* =  − 0.726, *P* < 0.0001).

We recruited 40 individuals with T2DM to receive 1.2 mg liraglutide for 4 months. Clinical characteristics of the 40 individuals before and after the 4-month liraglutide treatment (termed L0 and L4, respectively) are presented in Table S1. As baseline HbA1c is a major predictor in blood glucose therapies, we assessed the relationship between baseline HbA1c and HbA1c change using linear regression analysis. The result indicated that baseline HbA1c explains 52.7% of liraglutide treatment response variation (*R*^2^ = 0.527, *β* = −0.726, *P* < 0.0001, Fig. 1). In addition, BMI, HbA1c, homeostasis model assessment of insulin resistance (HOMA-IR), fasting blood glucose, 2-hour postprandial blood glucose, total cholesterol, triglycerides, HDL-C, and LDL-C were significantly lower in the post-liraglutide-treatment group than the pre-liraglutide-treatment groupusing Wilcoxon signed–rank test or *t*-test . However, there were no notable differences observed in fasting insulin, serum creatinine and the blood urea nitrogen between the two groups (Table S2).

Furthermore, due to many clinical characteristics may be associated with baseline HbA1c, adjusted for baseline HbA1c was suggested to avoid erroneous treatment response. So we used multiple linear regression to analyze the association between the baseline HbA1c and the baseline covariates (11 clinical characteristics), and the association between the baseline covariates and liraglutide treatment response after with or without adjustment for baseline HbA1c. However, the results indicated that none clinical characteristics were associated with both baseline HbA1c and liraglutide treatment response after without adjustment for baseline HbA1c. Only the blood urea nitrogen was associated with liraglutide treatment response after adjustment for baseline HbA1c (Table S3).

### Characteristics of the pyrosequencing results

To characterize the effects of liraglutide on the gut microbiome, we performed high-throughput sequencing of the V4 regions of 16S rRNA genes from the 80 fecal samples collected from the two groups. A total of 8,822,088 high-quality sequences and 7488 OTUs (97% similarity) were obtained from the 80 samples, and the average number of reads was 100, 626 and OTUs was 296 for each individual (Table S4). The reads/OTUs were assigned to 34 different phyla, and the top 3 dominant bacterial phyla of the two groups were Firmicutes, Bacteroidetes and Proteobacteria.

### Liraglutide modifies gut microbiota alpha and beta diversity in the T2DM

**Figure 2:**
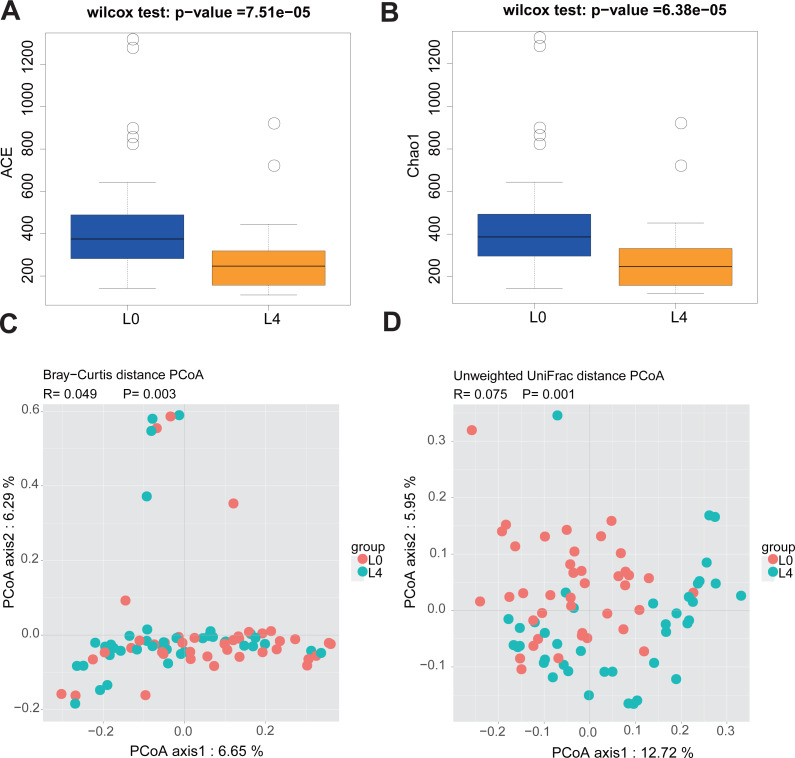
Liraglutide treatment affected the microbiota alpha diversity and beta diversity. (A–B) ACE and Chao1 were used to analyze the alpha diversity. Statistic analysis was performed by Wilcoxon rank-sum test, ∗*P* < 0.05. (C–D) PCoA of fecal microbiota from the two groups of patients using a Bray-Curtis distance matrix (C) and unweighted UniFrac distances (D). Statistical analysis was performed by ANOSIM test, ∗*P* < 0.05.

Since the participants have different duration of T2DM, we divided the 40 T2DM patients into three diabetes duration groups (short-duration group (<5 years); medium- duration group (5-10 years) and long-duration group (≥10 years)) and analyzed alpha and beta diversity in gut microbiota of the three groups. Alpha diversity is an index reflecting the variety of microbial species in stool samples and we also examined the patterns of beta diversity by calculating the dissimilarity in the composition of gut microbial (Rapacciuolo et al., 2019). The results indicated that the three duration groups had no significant difference in gut microbiota alpha and beta diversity (Figs. S1 and S2). Therefore, being treated with metformin for 2 months do not alter the fecal microbiome in T2DM patients. In order to evaluate the effect of liraglutide treatment on gut microbiota diversity, we analyzed the 80 fecal samples collected from pre- and post-liraglutide-treatment groups. The alpha diversity indices (the ACE, Chao1, Shannon and Simpson index) were used to describe alpha diversity. The community richness (ACE and Chao1) was significantly different between the two groups (Figs. 2A and 2B), showing a lower abundance in the post-liraglutide-treated group. However, there were no significant difference in the community diversity (Shannon and Simpson) of the two groups (Figs. S3A and S3B). As to the beta diversity, a principal-component analysis (PCoA) of a nonmetric multidimensional scaling plot (on a Bray-Curtis distance matrix) and unweighted UniFrac distances revealed significant qualitative differences in gut microbial community structure between the two groups (Figs. 2C and 2D), but there was no difference in weighted UniFrac distances (Fig. S3C).

### Liraglutide alters gut microbiota composition

**Figure 3:**
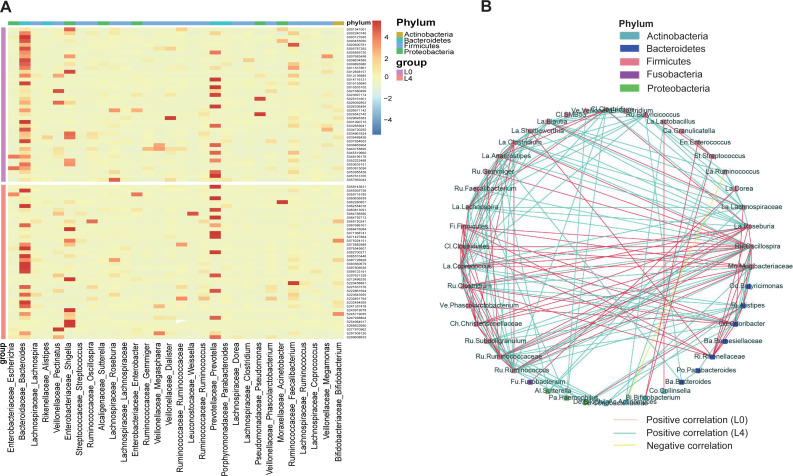
Liraglutide treatment promoted rapid changes in the composition of the gut microbiota. (A) Heat map showing changes in the abundance of bacterial strains before (L0) and after the 4-month liraglutide treatment (L4) (only the top 30 genera are listed here). (B) Genus–genus coabundance network of the pre- (L0) and the post-liraglutide-treatment group (L4). The edges indicate Spearman correlations of *r* > 0.5 or *r* <  − 0.5 and ∗*P* < 0.05 between the genera present in at least 60% samples.

To assess the effects of liraglutide on gut microbiota composition, a taxonomy-based comparison was performed and a broad overview of our taxonomic data from the 80 samples was given in Fig. 3A. A heat map was constructed to visualize the top 30 genus in the two groups (Fig. 3A). We also conducted a coabundance network analysis to investigate how different gut bacteria could interact with each other. The results indicated that 4 months of liraglutide treatment promoted an increased number of positive correlations among microbial genera, especially those within Firmicutes and Bacteroidetes. Negative correlation was identified in *Ruminococcus* (Firmicutes) and *Actinomyces* (Actinobacteria) (Fig. 3B).

### Phylogenetic and taxonomic profiles of gut microbiota

To analyze the statistical differences in microbial communities between the pre- and post-liraglutide-treatment of T2DM, the significant differences based on genomic characteristics were further confirmed by LEfSe analysis, which used LDA coupled with effect size measurement to identify bacterial taxa with sequences that differed in the abundance of the two groups.

**Figure 4:**
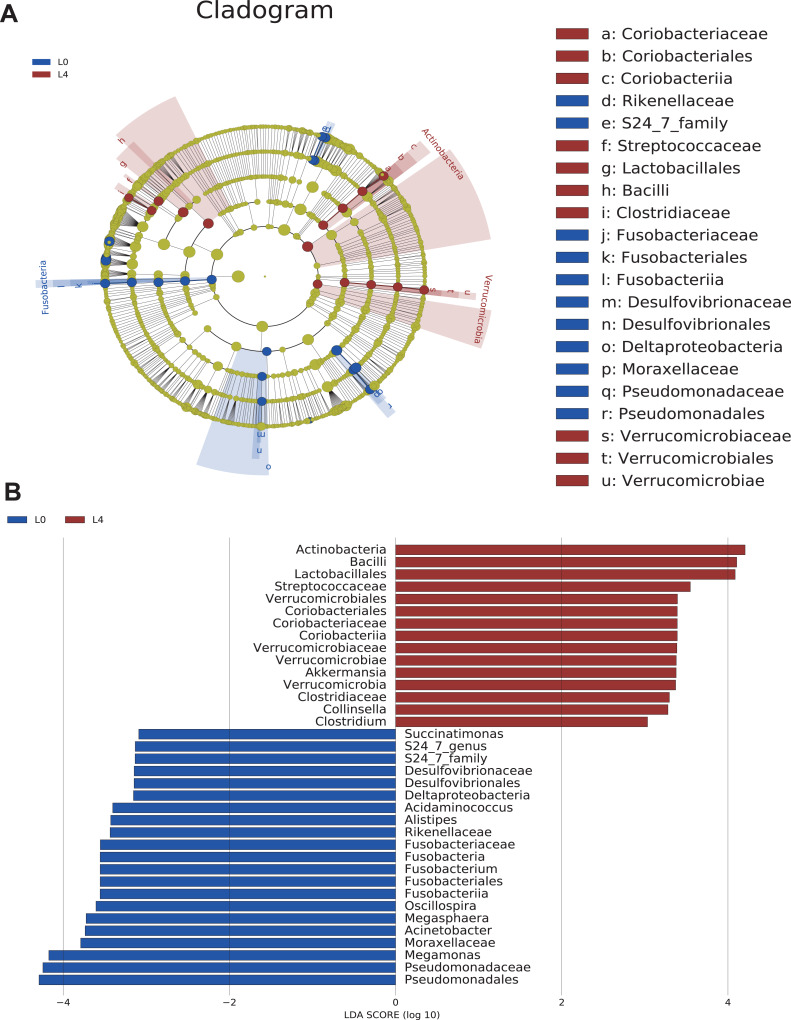
The overall structure and composition of the gut microbiota difference between the pre- and post-liraglutide-treatment group. (A) The cladogram depicts the phylogenetic distribution of microbial lineages in fecal samples taken before (L0) and after the 4-month liraglutide treatment (L4). Differences in abundant microbiota are listed and distinguished by different color (blue for L0 group and brown for L4 group). (B) Key phylotypes of the gut microbiota responding to liraglutide treatment. The histogram shows the lineages with LDA values >3.0 and with a *P < 0.05 as determined by LEfSe analysis.

Cladograms were obtained by the LEfSe analysis, which showed the most significantly difference at taxonomic levels between the two groups. The size of each circle represents the abundance of certain bacteria (Fig. 4A).

Upon analyzing the fecal samples, 36 phylotypes were identified as high-dimensional biomarkers (Table S5). Twenty-one species of bacteria were abundant in the pre-liraglutide-treatment group and 15 species were abundant in the post-liraglutide-treatment group. At the phylum level, the abundance of Fusobacteria was significantly higher in the pre-liraglutide-treatment group, while Verrucomicrobia and Actinobacteria were significantly higher in the post-liraglutide-treatment group. At the genus level, nine genera were found in significantly high abundances in the pre-liraglutide-treatment group. These genera included *Acinetobacter* (*p* = 0.016), *Oscillospira* (*p* = 0.013), *Acidaminococcus* (*p* = 0.021), *Succinatimonas* (*p* = 0.042), *S24_7* (*p* = 0.008), *Megamonas* (*p* = 0.0005), *Alistipes* (*p* = 0.035), *Fusobacterium* (*p* = 0.017) and *Megasphaera* (*p* = 0.0002). The genera *Collinsella* (*p* = 0.011), *Akkermansia* (*p* = 0.002) and *Clostridium* (*p* = 0.002) were enriched in the post-liraglutide-treatment group (Fig. 4B).

### Correlation between clinical features and gut microbiota

According to the LDA analysis (LDA value > 3.0), we discovered the relative abundance of 12 bacterial genera (Table S5) between the two groups. To investigate the inter associations between gut microbiota and clinical status of the host, we identified the statistical correlations between the 12 genera and the 11 clinical features, including age, sex, BMI, family history of diabetes, diabetes duration, family history of cardiovascular disease, family history of diabetic nephropathy (DN), family history of diabetic retinopathy (DR), family history of diabetic peripheral neuropath (DPN), smoking status and alcoholism status (Table S6).

**Figure 5:**
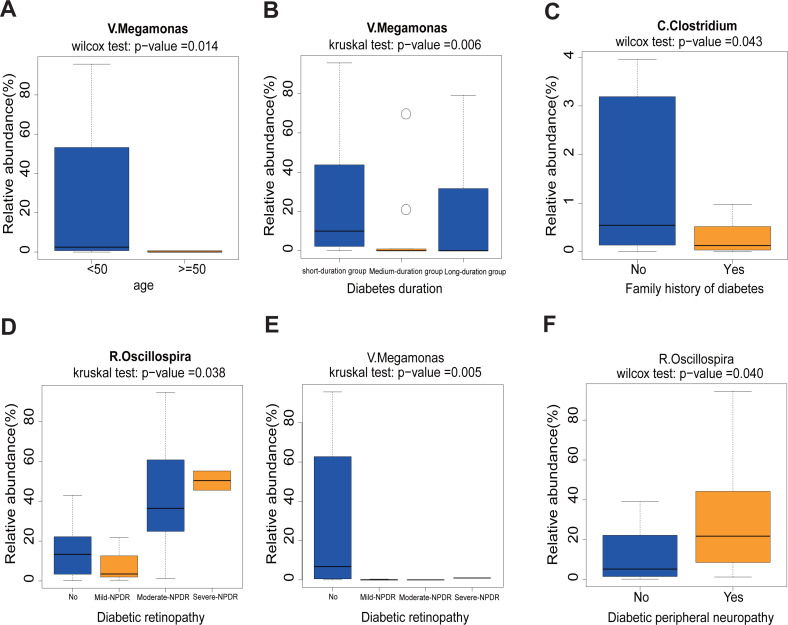
Correlation analysis between 11 clinical features and 12 genera. (A) Comparison of 12 relative abundance genera between <50 age and ≥50 age group. Obviously, *Megamonas* was lower in the ≥50 age group. (B) The abundance of *Megamonas* was lower in the medium- and long-duration groups. (C) The abundance of *Clostridium* was lower in the family history of diabetes group. (D–E) The abundance of *Oscillospira* was higher while the abundance of *Megamonas* was lower in the DR group. (F) The abundance of *Oscillospira* was higher in the DPN group. Statistical analysis was performed by Wilcoxon rank-sum test or Kruskal-Wallis test, ∗*P* < 0.05.

First, we found that the abundance of *Megamonas* was significantly lower in the older age group (≥50) (*p* = 0.014), medium- and long-duration groups (*p* = 0.006) (Figs. 5A and 5B). What’s more, the levels of *Clostridum* genus was lower in the family history of diabetes (*p* = 0.043) group (Fig. 5C). *Oscillospira was* significantly different between the Non-DR group and DR groups, in which severe non-proliferative DR (NPDR) sub-group had the highest abundance of *Oscillospira* (*p* = 0.038) (Fig. 5D). We also found that the abundance of *Megamonas* was lower in the DR group (*p* = 0.005) (Fig. 5E). Last but not least, the higher abundance of *Oscillospira* was found in the DPN group, but not in the non-DPN group (*p* = 0.040) (Fig. 5F).

### Effect of liraglutide-treatment administration on metabolic pathways

**Figure 6:**
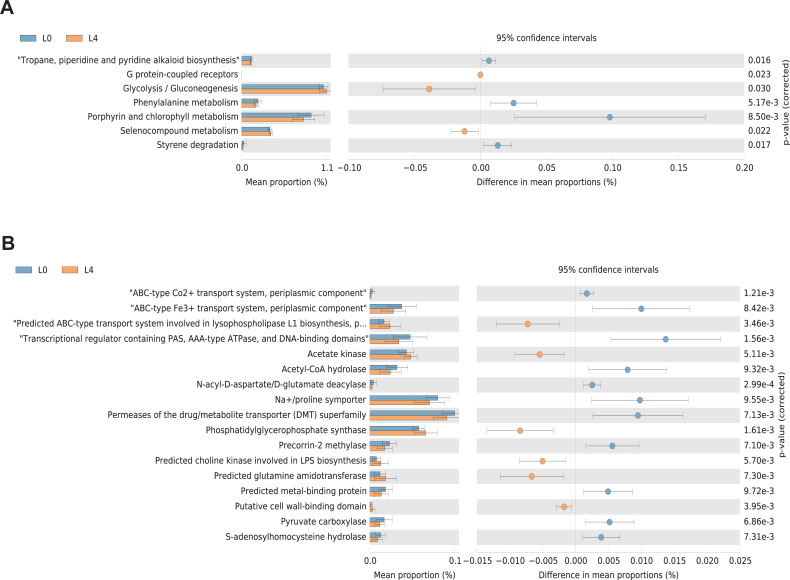
Differential PICRUSt-predicted KEGG and COG pathways in individuals before (L0) and after the 4-months liraglutide treatment (L4). The KEGG (A) and COG (B) pathways demonstrating seven and 17 significant differences between the two groups, respectively. For each comparison in predicted KEGG or COG pathways, the mean proportion and the 95% CI difference in mean proportions are also illustrated. Statistical analysis was performed by Welch’s *t*-test, ∗*P* < 0.05.

KEGG and COG pathway comparisons were performed to explore potential differences in the functional composition of the microbiome between the pre- and post-liraglutide-treatment group. Figure 6 highlights the significant differences in the distribution of metabolic pathways, and compares the microbiota functions between the two groups. As listed in Fig. 6A, seven differences were observed in KEGG pathways. Three KEGG pathways (including G protein-coupled receptors, Glycolysis/Gluconeogenesis and Selenocompound metabolism) were enriched in the post-liraglutide-treatment group, and 4 KEGG pathways (including tropane, piperidine and pyridine alkaloid biosynthesis, phenylalanine metabolism, porphyrin and chlorophyll metabolism, and styrene degradation) were enriched in the pre-liraglutide-treatment group.

As shown in Fig. 6B, 17 different functional COG pathways between the two groups were found. Six of them were enriched in the post-liraglutide-treatment group, including predicted ABC-type transport system involved in lysophospholipase L1 biosynthesis, acetate kinase, phosphatidylglycerophosphate synthase, predicted choline kinase involved in lipopolysaccharides (LPS) biosynthesis, predicted glutamine amidotransferase and putative cell wall-binding domain. In contrast, the pre-liraglutide-treatment group were enriched in ABC-type Co2+ transport system, periplasmic component, ABC-type Fe3+ transport system, periplasmic component, transcriptional regulator containing PAS, AAA-type ATPase, and DNA-binding domains, acetyl-CoA hydrolase, N-acyl-D-aspartate/D-glutamate deacylase, Na+/proline symporter, permeases of the drug/metabolite transporter (DMT) superfamily, precorrin-2 methylase, predicted metal-binding protein, pyruvate carboxylase and s-adenosylhomocysteine hydrolase.

## Discussion

T2DM is a complex metabolic disorder and public health issue throughout the world. It is also one of the most severe chronic diseases that affect human health. According to the estimation, the prevalence of diabetes may increase 69% in developing countries and 20% in developed countries between 2010 and 2030 (Shaw, Sicree & Zimmet, 2010). The metabolic potential of the gastrointestinal tract and its microbiota are increasingly recognized as promising targets to improve T2DM treatment (Brunkwall & Orho-Melander, 2017). Thus, there is an urgent need in the development of more efficient prevention and treatment strategies.

As a GLP-1 analogue, liraglutide has shown the therapeutic effects by changing the structural of gut microbiota in diabetic male rats (Zhang et al., 2018). In this study, although all participants were stable on metformin as a monotherapy for 2 months before the recruitment, neither alpha nor beta diversity was different among the three diabetes duration groups. Then all 40 T2DM patients were switched from oral metformin to subcutaneous injections of liraglutide for 4 months. We found that the liraglutide treatment lowered BMI, blood glucose, HbA1c, HOMA-IR and relieved dyslipidemia in T2DM patients. Similarity, in a diabetic rat study, insulin level and HOMA-IR were significantly decreased (Zhang et al., 2018), and blood glucose and body weight were much lower after being treated with liraglutide (Wang et al., 2016). In the present study, we found that baseline HbA1c is associated with liraglutide treatment response. As clinical characteristics may be associated with baseline HbA1c, many T2DM studies have adjusted for baseline HbA1c to avoid incorrect treatment response using linear regression method (Jones et al., 2016; Thong et al., 2014). In this study, neither baseline HbA1c nor liraglutide treatment response after without adjustment for baseline HbA1c was associated with baseline covariates. However, the blood urea nitrogen was associated with treatment response after adjustment for baseline HbA1c. Many studies have indicated that blood urea nitrogen is associated with increased an risk of insulin use in diabetes mellitus (Xie et al., 2018a; Xie et al., 2018b). Jones et al. reported that false positive associations may arise and true associations may be covered up by negative results if without adjustment for baseline HbA1c (Jones et al., 2016). In this study, without adjustment for baseline HbA1c, the blood urea nitrogen was not associated with treatment response; while adjustment for baseline HbA1c, the blood urea nitrogen was associated with treatment response. Furthermore, we analyzed the effects of liraglutide on the gut microbiota of the 80 samples collected from the pre- and post-liraglutide-treatment groups. Several interesting results were observed. First of all, at the phyla level, Firmicutes, Bacteroidetes and Proteobacteria were the top 3 dominant bacterial phyla between the two groups. A previous study reported that Bacteroidetes and Proteobacteria, could produce LPS, and subsequently trigger inflammatory response and contribute to the development of diabetes (Larsen et al., 2010). The diabetic rat microbiome was profoundly dominated by the phyla Firmicutes and Bacteroides, while liraglutide-treated rat group had a reduced Firmicutes/Bacteroidetes ratio and relative abundance of Tenericutes (Zhang et al., 2018). According to our taxonomic data from the 80 samples, patients who had enriched genus *Bacteroides* usually had low abundance of genus *Prevotella*, which is consistent with the previous study performed in colorectal cancer (Yachida et al., 2019). Zhang et al. reported that *Prevotella* genera was much more lower in liraglutide-treated rats (Zhang et al., 2018). Secondly, by using a Bray-Curtis distance matrix and unweighted UniFrac PCoA analysis, we confirmed that two major components in the gut microbiota of the two groups were structurally separated from each other. What’s more, according to LEfSe analysis, 9 genera including *Acinetobacter*, *Oscillospira*, *Acidaminococcus*, *Succinatimonas*, *S24_7*, *Megamonas*, *Alistipes*, *Fusobacterium* and *Megasphaera* were found in significantly high abundances in the pre-liraglutide-treatment group. In a previous study, Li et al. reported that *Acinetobacter* was significantly abundant in T2DM group than that in healthy subjects (Li et al., 2019). *Oscillospira* was reported to be significantly abundant in high-fat diet mice group than in a chow diet mice group (Wu et al., 2019). However, *Fusobacterium* genera in fecal samples were not significantly different between the T2DM and healthy people (Sedighi et al., 2017). Therefore, large studies were needed to verify this issue in near future. According to our research, *Megamonas* was significantly lower in the older group, medium- and long- duration group, which is in accord with the study that *Megamonas* was significant lower in longevity village communities than urbanized town communities (Park et al., 2015). A similar study indicated that the abundance of *Alistipes* was significantly increased in rat of T2DM (Zhu et al., 2020). *Clostridum* was lower in the T2DM family history of diabetes group, similarly, *Clostridum* was significantly lower in the fecal samples of diabetic patients than in those of control subjects in Japanese (Yamashiro, 2017). Another study further indicated that *Clostridium* was negatively correlated with fasting blood glucose, HbA1c and insulin levels (Karlsson et al., 2013).

On contrary, liraglutide-treated group had an increased level of *Collinsella*, *Akkermansia* and *Clostridium* at the genus level. In a pre-clinical study, the abundance of *Akkermansia* was increased by the metformin treatment in the mice fed by high fat diet (HFD), and oral administration of *Akkermansia* enhanced glucose tolerance and attenuated adipose tissue inflammation in HFD-fed mice (Shin et al., 2014). Another study reported the low abundance of *Clostridium* in European women with T2DM, as well as the negative correlation of *Clostridium* with fasting blood sugar, glycated hemoglobin, insulin, and plasma triglycerides (Karlsson et al., 2013). In our study, after being treated with liraglutide for 4 months, the abundance of *Clostridium* was decreased in the family history of diabetes group, which is consistent with the findings that the abundance of *Clostridium* is lower in diabetic patients (Yamashiro, 2017). In the diabetic rat liraglutide-treated study, LEfSe analysis showed 11 bacteria significantly difference between the liraglutide-treated group and diabetic group. The genera Flavonifractor and Lachnoclostridium, species Ruminococcus_gnavus, Flavonifractor_plautii, and Bacteroides_acidifaciens were much more abundant in the liraglutide-treated group than the diabetic group. The reduced bacteria were mainly in the family Christensenellaceae, genera Christensenellaceae_R_7_group, the genera Ruminococcaceae_UCG_010, Ruminoclostridium_6, and Prevotella_9, and the class Mollicutes (Zhang et al., 2018). These results indicated that microbiome structures of rat and human were mainly different.

Finally yet importantly, the PICRUSt analysis revealed 7 different KEGG pathways and 17 COG pathways. KEGG analysis further indicated that these gut bacteria in T2DM were strongly associated with the dysregulation of several metabolic processes such as glycolysis/gluconeogenesis metabolism; phenylalanine metabolism; porphyrin and chlorophyll metabolism; and selenocompound metabolism. For example, phenylalanine metabolism, showed lower abundance in the post-liraglutide-treatment group. It is reported that phenylalanine was associated with insulin resistance, and may increase the risk of T2DM (Guasch-Ferre et al., 2016). Glycolysis/gluconeogenesis pathway was higher in the post-liraglutide-treatment group, which is consistent with the findings that glycolysis/gluconeogenesis related metabolites is significantly associated with T2DM risk in a Mediterranean population (Guasch-Ferre et al., 2020). In addition, we found 17 different functional COGs between the two groups. For example, periplasmic component, ATPase component and permeases of the drug/metabolite transporter (DMT) were enriched in the pre-liraglutide-treatment group, which promote glucose and ribose/galactoside using to regulate energy according to the previous study (Liu et al., 2019). Considering the fact that T2DM is a metabolic disease and several metabolic disorder phenotypes were observed in T2DM patients, we speculated that gut microbiota affects the host via metabolites, which provides a further understanding between the microbiome–metabolome interaction in T2DM and may be helpful for the diagnosis and treatment of T2DM in the future.

There were still several limitations in this study. Specifically, the sample size was relatively small to represent the combined effects of T2DM and liraglutide. Besides, we did not recruit a 4-month-metformin-treated group to compare with the current- liraglutide-treated group. Although our collected clinical characteristics were significant changes between the pre- and post-liraglutide treatment groups. While adjustment for baseline HbA1c, only blood urea nitrogen was associated with liraglutide treatment response in the 40 T2DM patients. Therefore, it is urgent to enlarge cohort to verify this issue in the future. Due to the extremely complicated interactions between gut microbiota and host, as well as our knowledge and research capability, the potential mechanisms behind this interaction were not further explored. Meanwhile, metagenomic sequencing has been widely employed to explore novel changes in the functional potential of the microbiota. However, in the present study, the results of prediction COG and KEGG functional analysis were analyzed by the method of 16S rRNA gene sequencing. Besides, due to the small sample size, we only covered part of the correlations between gut microbiota and clinical features. Therefore, to fully elucidate the modifiable capacity of the gut microbiota and its potential applications in the prevention and treatment for type 2 diabetes, large longitudinal, interventional studies and multicenter strategies are further required.

In conclusion, our findings suggest that liraglutide treatment is associated with baseline HbA1c, blood urea nitrogen and gut microbiota composition in T2DM patients. This may contribute to the beneficial effects of liraglutide against diabetes. In particular, the phenylalanine metabolism, porphyrin and chlorophyll metabolism and selenocompound metabolism pathways were predicted by KEGG pathways without no functional analysis in T2DM yet. So further researches are required to elucidate the mechanisms by which liraglutide affects the gut microbiota of diabetes patients, and this may serve as potential therapeutic targets for T2DM.

##  Supplemental Information

10.7717/peerj.11128/supp-1 Figure S1 Microbiota alpha diversity was performed in the three diabetes duration groups (A-D) ACE, Chao1, Shannon and Simpson index were used to analysis the alpha diversity.

10.7717/peerj.11128/supp-2 Figure S2 Microbiota beta diversity was performed in the three diabetes duration groups (A–C) PCoA of fecal microbiota from the three groups of individuals using a weighted UniFrac distances, unweighted UniFrac distances and Bray-Curtis distance matrix.

10.7717/peerj.11128/supp-3 Figure S3 Liraglutide treatment affected the microbiota alpha diversity and beta diversity (A-B) Shannon and Simpson index were used to analysis the alpha diversity. (C) PCoA of fecal microbiota from the two groups of individuals using a weighted UniFrac distances.

10.7717/peerj.11128/supp-4 Table S1 Clinical characteristics of 40 individuals raw data

10.7717/peerj.11128/supp-5 Table S2 Clinical characteristics for the 40 individuals with T2DM enrolled in this study

10.7717/peerj.11128/supp-6 Table S3 The associations between baseline covariates and HbA1c change after liraglutide therapy without or with adjustment for baseline HbA1c

10.7717/peerj.11128/supp-7 Table S4 A summary of the pyrosequencing data

10.7717/peerj.11128/supp-8 Table S5 36 phylotypes were identified by LDA analysis

10.7717/peerj.11128/supp-9 Table S6 Clinical characteristics for the 40 individuals with T2DM enrolled in this study

## Additional Information and Declarations

### Data Availability

The following information was supplied regarding data availability: Data are available at the NCBI BioProject database: PRJNA665123.
