# Supplementary figures and images for: Liraglutide-induced structural modulation of the gut microbiota in patients with type 2 diabetes mellitus
Source: PeerJ. 2021 Apr 1;9:e11128. doi: 10.7717/peerj.11128 (PMC8019531; doi:10.7717/peerj.11128)

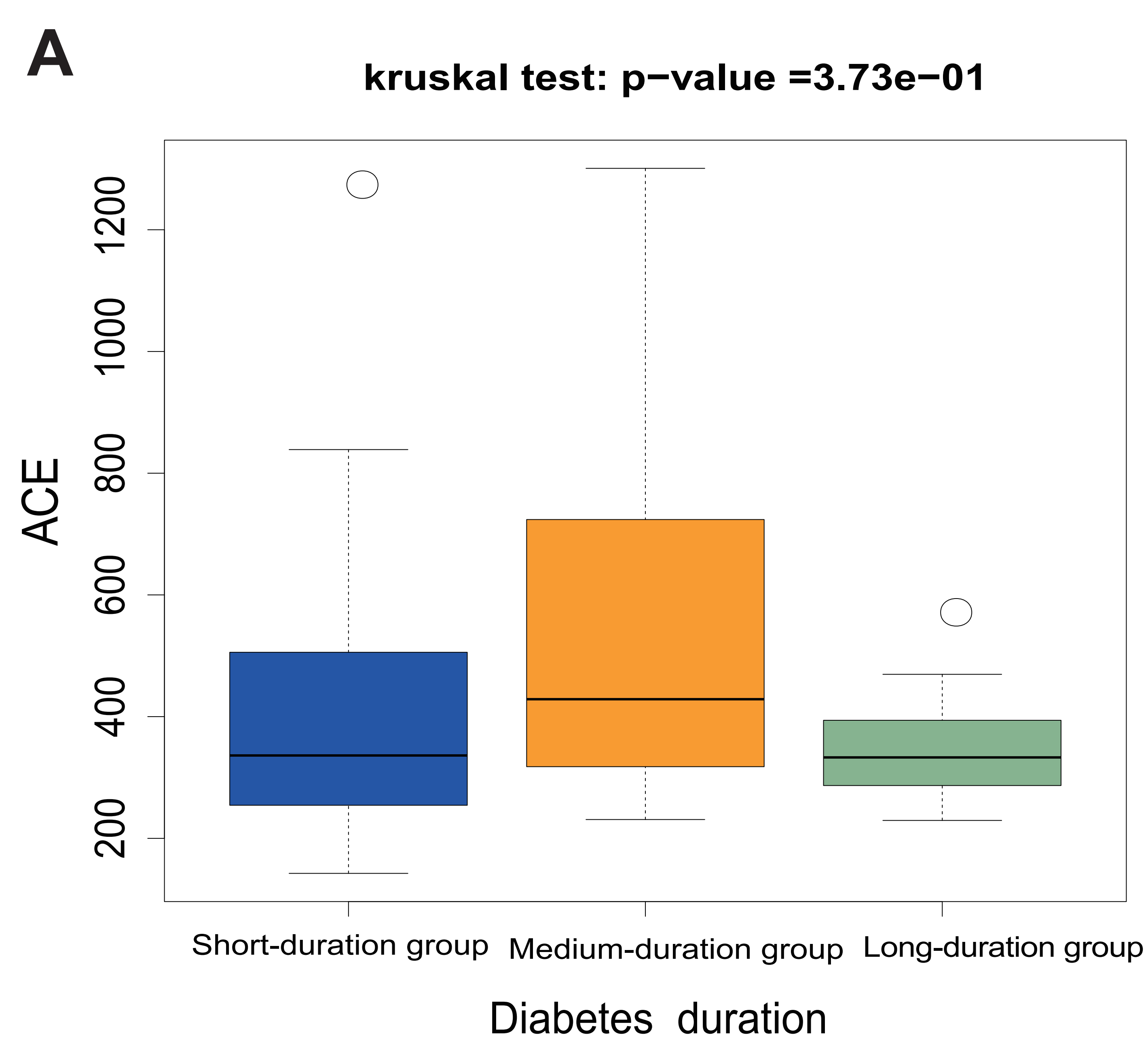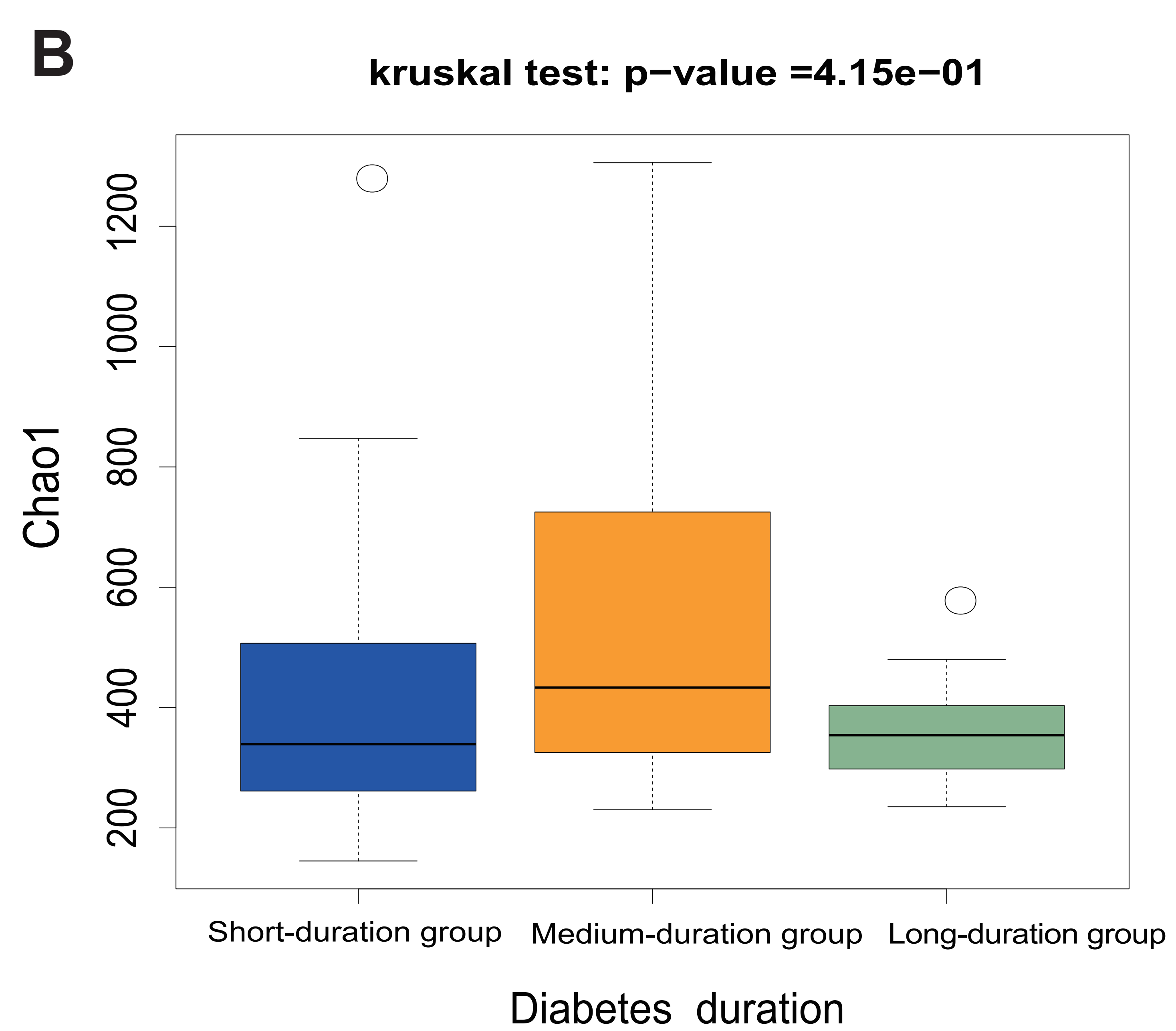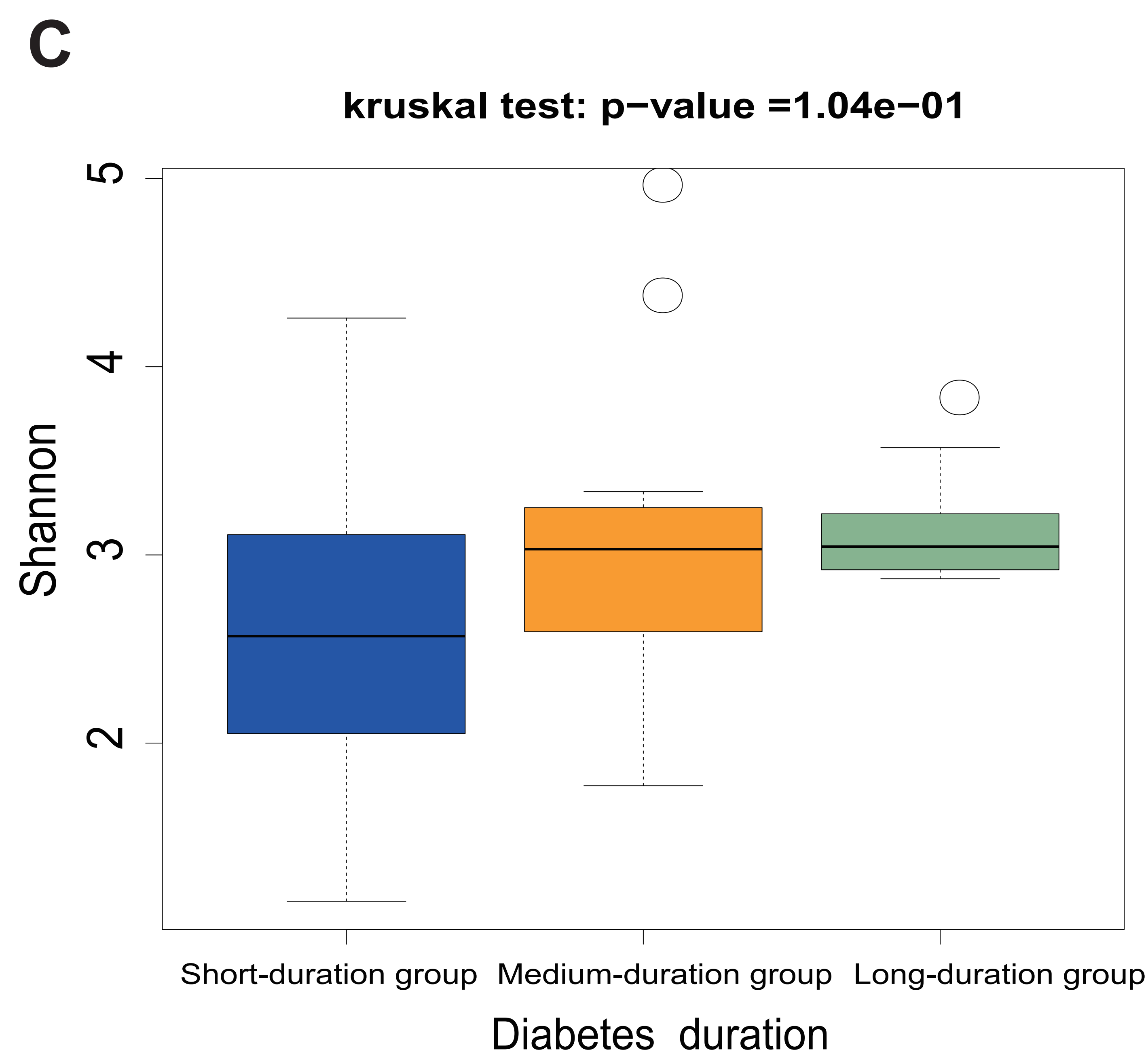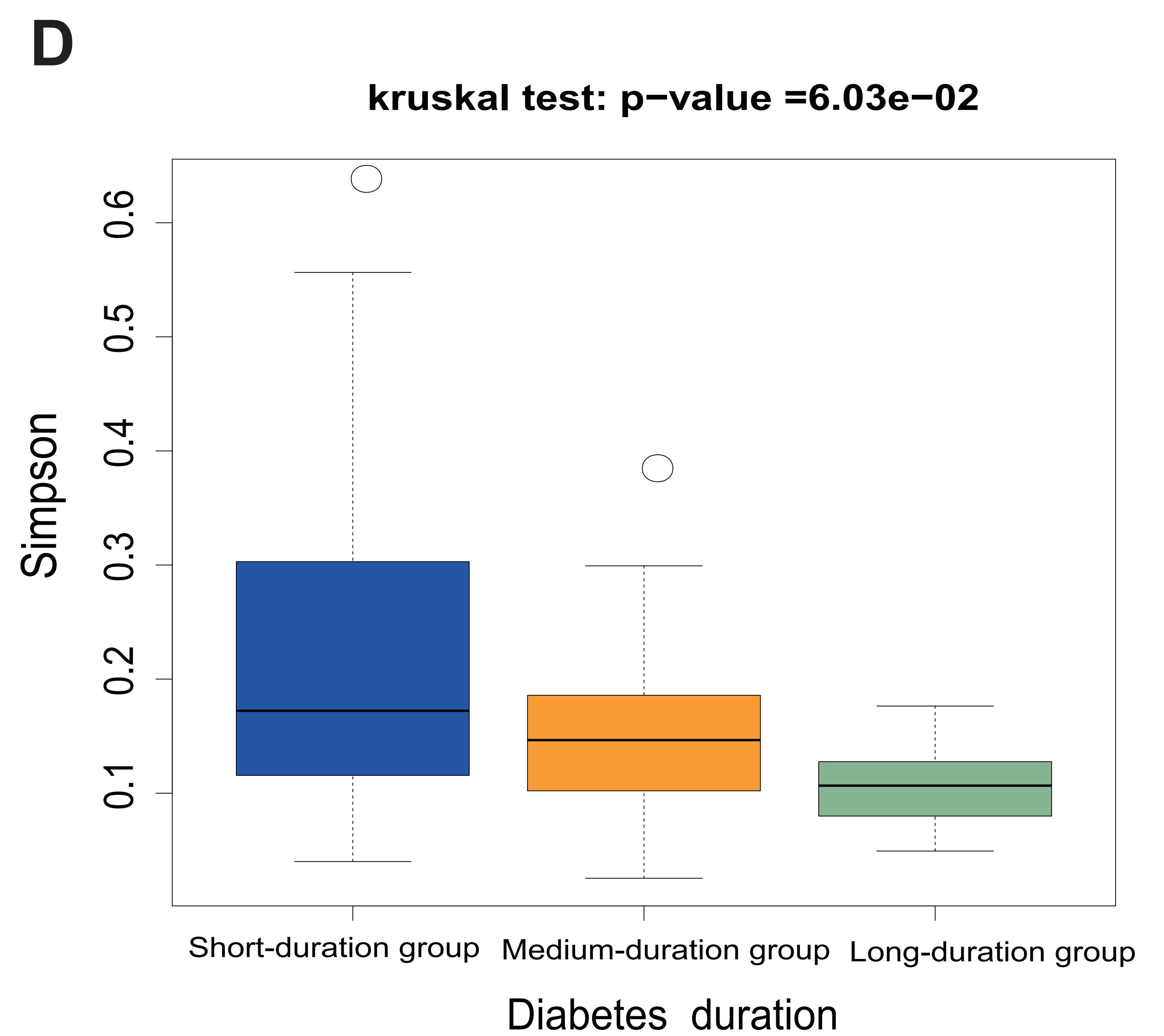

Supplement: Figure S1 — (A-D) ACE, Chao1, Shannon and Simpson index were used to analysis the alpha diversity. [file peerj-09-11128-s001.pdf]

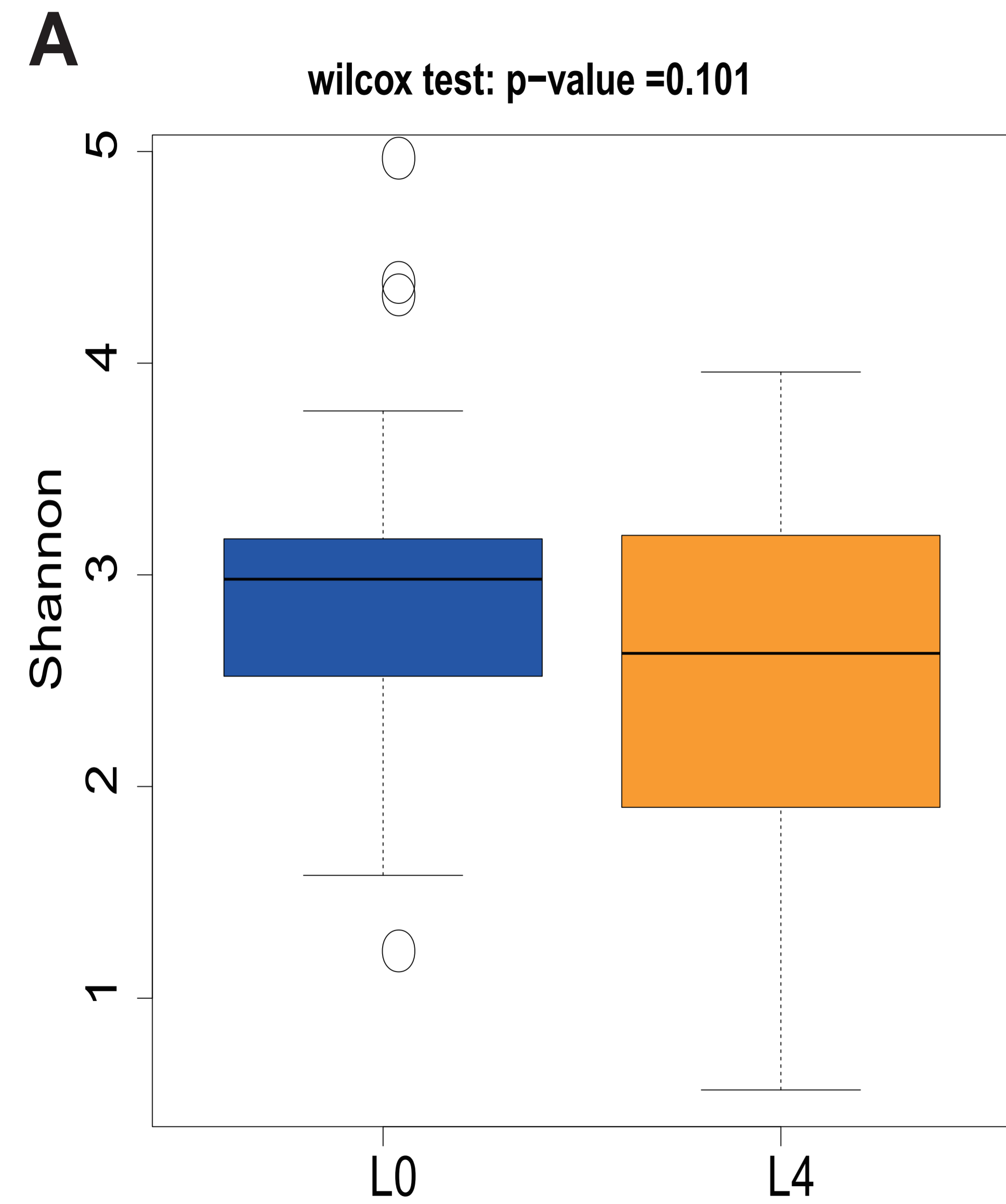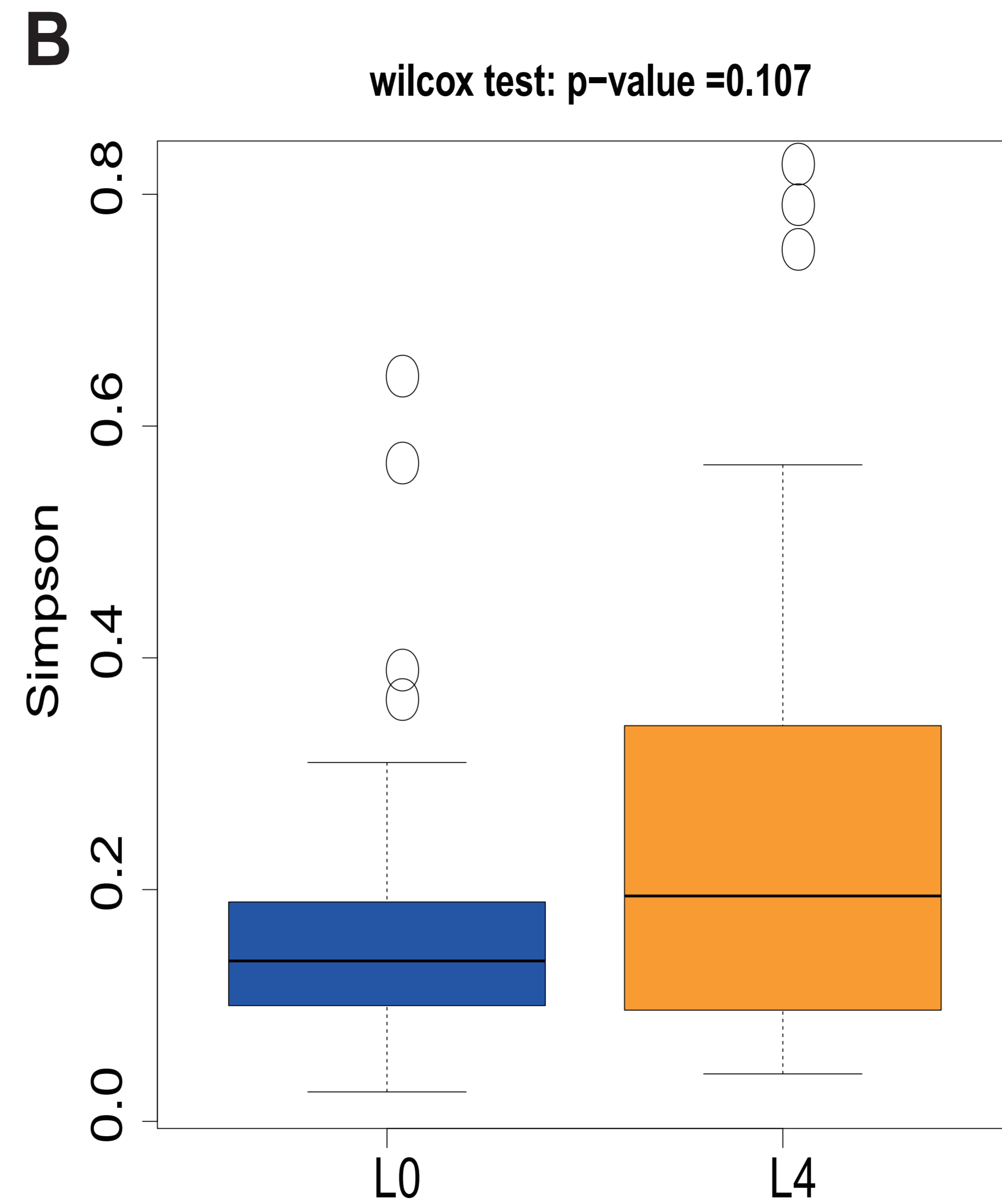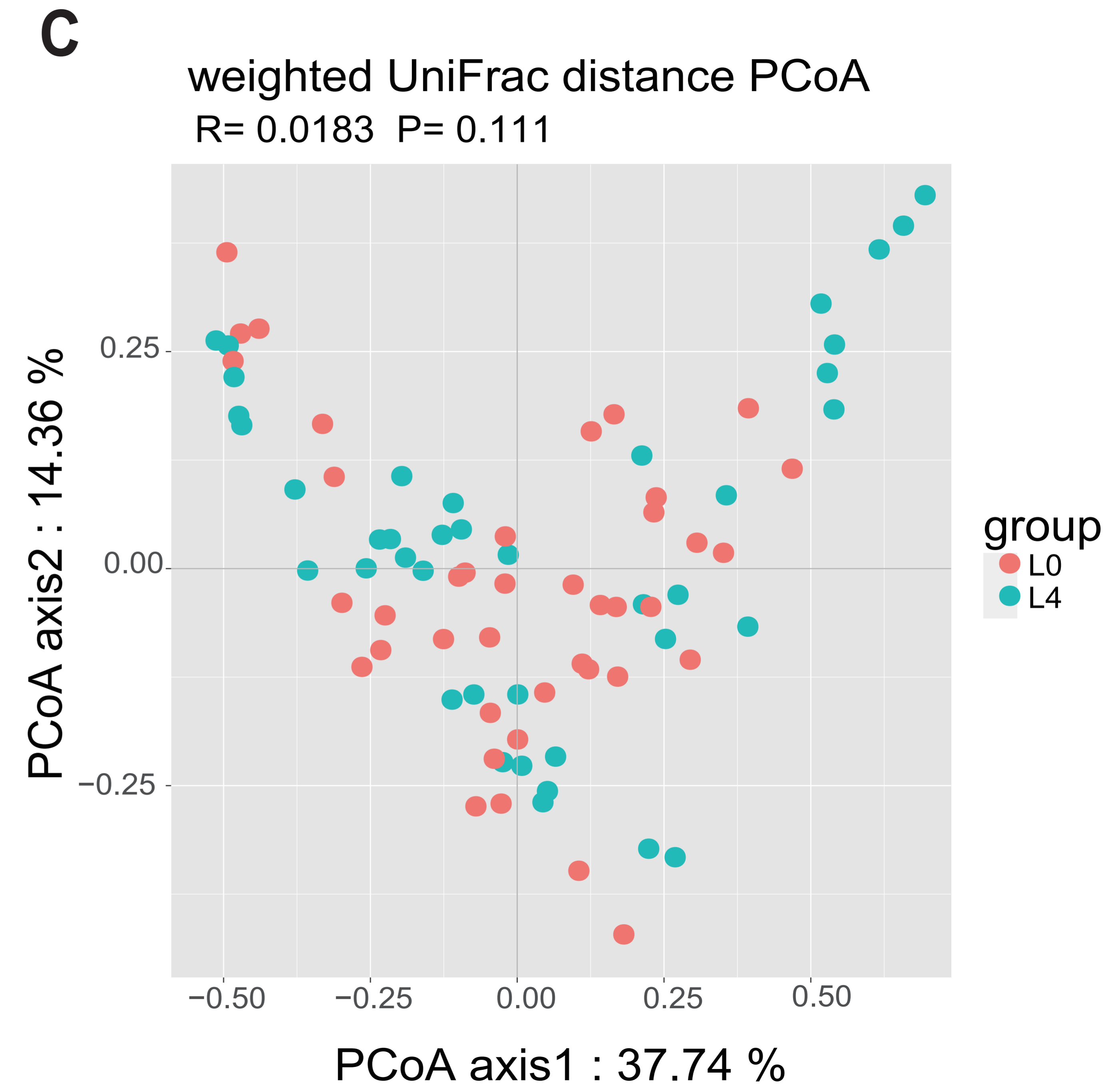

Supplement: Figure S3 — (A-B) Shannon and Simpson index were used to analysis the alpha diversity. (C) PCoA of fecal microbiota from the two groups of individuals using a weighted UniFrac distances. [file peerj-09-11128-s003.pdf]
