# Supplementary material for: Liraglutide-induced structural modulation of the gut microbiota in patients with type 2 diabetes mellitus
Source: PeerJ. 2021 Apr 1;9:e11128. doi: 10.7717/peerj.11128 (PMC8019531; doi:10.7717/peerj.11128)
Supplement: Figure S2 — (A–C) PCoA of fecal microbiota from the three groups of individuals using a weighted UniFrac distances, unweighted UniFrac distances and Bray-Curtis distance matrix. [file peerj-09-11128-s002.pdf]

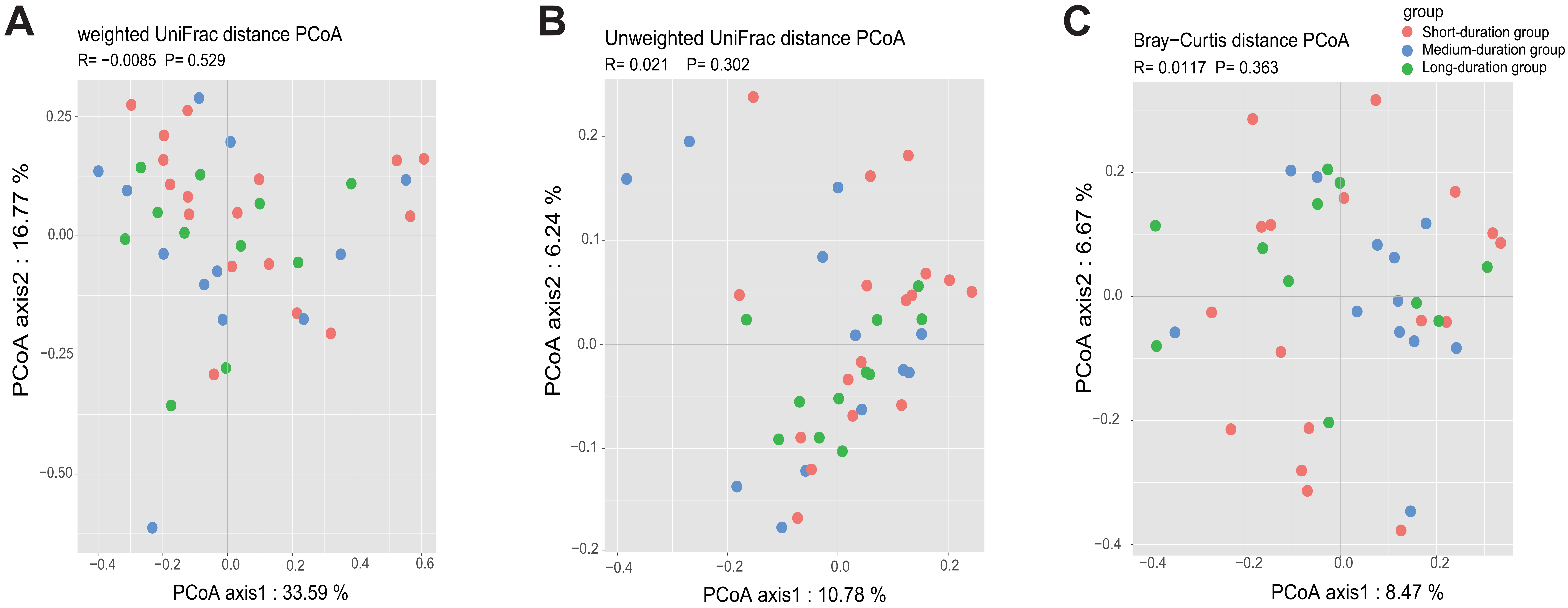

A
weighted UniFrac distance PCoA
R= −0.0085 P= 0.529
PCoA axis2 : 16.77 %
PCoA axis1 : 33.59 %
0.25
0.00
−0.25
−0.50
−0.4
−0.2
0.0
0.2
0.4
0.6
B
Unweighted UniFrac distance PCoA
R= 0.021 P= 0.302
PCoA axis2 : 6.24 %
PCoA axis1 : 10.78 %
0.2
0.1
0.0
−0.1
−0.2
−0.4
−0.2
0.0
0.2
C
Bray−Curtis distance PCoA
R= 0.0117 P= 0.363
PCoA axis2 : 6.67 %
PCoA axis1 : 8.47 %
0.2
0.0
−0.2
−0.4
−0.4
−0.2
0.0
0.2
group
Short-duration group
Medium-duration group
Long-duration group
