# Supplementary material for: Liraglutide-induced structural modulation of the gut microbiota in patients with type 2 diabetes mellitus
Source: PeerJ. 2021 Apr 1;9:e11128. doi: 10.7717/peerj.11128 (PMC8019531; doi:10.7717/peerj.11128)
Supplement: Table S2 [file peerj-09-11128-s005.docx]

| Table S2. Clinical characteristics for the 40 individuals with T2DM enrolled in this study. | | | |
| --- | --- | --- | --- |
|  | L0 ( n=40) | L4 (n=40) | *P* |
| Age (years) | 47 (25-83) | - |  |
| Sex (male/female) | 40 (31/9) | - |  |
| BMI (kg/m^2^) | 30.16±0.56 | 27.09±0.49 | <0.0001 |
| % HbA1c | 9.17 ±0.28 | 6.52±0.21 | <0.0001 |
| HbA1c (mmol/mol) | 76.67±3.02 | 47.78±2.31 | <0.0001 |
| Fasting insulin (pmol/mL) | 13.91±1.72 | 14.12±1.27 | 0.871 |
| HOMA-IR | 6.85±0.64 | 4.43±0.40 | 0.0002 |
| Fasting blood glucose (mmol/L) | 11.91±0.61 | 7.23±0.36 | <0.0001 |
| 2 hours postprandial blood glucose (mmol/L) | 16.35±0.70 | 9.51±0.37 | <0.0001 |
| Total cholesterol (mmol/L) | 5.20±0.23 | 4.22±0.16 | 0.0004 |
| Triglycerides (mmol/L) | 4.08±0.70 | 1.53±0.11 | <0.0001 |
| HDL-C (mmol/L) | 0.85±0.04 | 1.03±0.02 | 0.0002 |
| LDL-C (mmol/L) | 3.27±0.17 | 2.76±0.13 | 0.0067 |
| Serum creatinine(μmol/L) | 63.53±2.45 | 58.64±2.30 | 0.088 |
| Blood urea nitrogen (mmol/L) | 5.70±0.23 | 5.28±0.22 | 0.114 |

Values are expressed as means±SEM; BMI, body-mass index; HbA1c, hemoglobin A1c; HOMA-IR, homeostasis model assessment of insulin resistance; HDL-C, high-density lipoprotein cholesterol；LDL-C, low-density lipoprotein cholesterol；**P*<0.05 was considered to be significant.
