# Supplementary material for: Liraglutide-induced structural modulation of the gut microbiota in patients with type 2 diabetes mellitus
Source: PeerJ. 2021 Apr 1;9:e11128. doi: 10.7717/peerj.11128 (PMC8019531; doi:10.7717/peerj.11128)
Supplement: Table S3 [file peerj-09-11128-s006.docx]

Table S3. The associations between baseline covariates and HbA1c change after liraglutide therapy without or with adjustment for baseline HbA1c.

| Association (linear regression) | n | Association with baseline HbA1c | Association with HbA1c change (unadjusted by baseline HbA1c) | Association with HbA1c change (adjusted by baseline HbA1c) |
| --- | --- | --- | --- | --- |
| BMI (kg/m^2^) | 40 | β=-0.081  (-0.231, 0.068) p=0.274 | β=0.002  (-0.152, 0.155) p=0.982 | β=-0.040  (-0.179, 0.099) p=0.562 |
| Fasting blood glucose （mmol/L） | 40 | β=0.197  (-0.072, 0.465) p=0.145 | β=-0.266  (-0.541, 0.010) p=0.058 | β=-0.167  (-0.419, 0.088) p=0.191 |
| 2 hours postprandial blood glucose（mmol/L） | 40 | β=0.073  (-0.120, 0.267) p=0.445 | β=-0.041  (-0.239, 0.157) p=0.674 | β=-0.004  (-0.181, 0.174) p=0.965 |
| Fasting insulin（pmol/mL） | 40 | β=-0.027  (-0.136, 0.083) p=0.623 | β=0.007  (-0.105,0.119) p=0.901 | β=-0.007  (-0.106,0.093) p=0.893 |
| HOMA-IR | 40 | β=0.042  (-0.264, 0.348) p=0.782 | β=-0.038  (-0.351, 0.276) p=0.808 | β=-0.016  (-0.294, 0.262) p=0.905 |
| Total cholesterol (mmol/L) | 40 | β=-0.017  (-0.758, 0.723) p=0.963 | β=-0.297  (-1.055, 0.461) p=0.429 | β=-0.305  (-0.977, 0.366) p=0.359 |
| Triglycerides (mmol/L) | 40 | β=0.088  (-0.140, 0.317) p=0.436 | β=-0.076  (-0.310, 0.158) p=0.511 | β=-0.031  (-0.241, 0.178) p=0.762 |
| HDL-C (mmol/L) | 40 | β=1.618  (-1.491, 4.728) p=0.296 | β=-1.277  (-4.464, 1.909) p=0.419 | β=-0.455  (-3.334, 2.424) p=0.748 |
| LDL-C (mmol/L) | 40 | β=-0.093  (-1.034, 0.849) p=0.842 | β=0.091  (-0.874, 1.055) p=0.849 | β=0.044  (-0.811, 0.899) p=0.917 |
| Serum creatinine（μmol/L） | 40 | β=0.003  (-0.031, 0.037) p=0.854 | β=-0.018  (-0.053, 0.016) p=0.288 | β=-0.017  (-0.047, 0.014) p=0.272 |
| Blood urea nitrogen（mmol/L） | 40 | β=0.006  (-0.387, 0.399) p=0.976 | β=0.397  (-0.005, 0.799) p=0.053 | β=0.400  (0.043, 0.757) p=0.029* |

BMI, body-mass index; HbA1c, hemoglobin A1c; HOMA-IR, homeostasis model assessment of insulin resistance; HDL-C, high-density lipoprotein cholesterol；LDL-C, low-density lipoprotein cholesterol；β = linear regression β coefficient, standardised for baseline covariates to represent HbA1c response difference (in baseline HbA1c or change after treatment, %) for a 1 standard deviation increase in baseline covariate. A positive β suggests a smaller HbA1c reduction with a higher value of the baseline covariate. Numbers in brackets represent the 95% confidence interval around β. **P*<0.05 was considered to be significant.
