# Supplementary material for: Liraglutide-induced structural modulation of the gut microbiota in patients with type 2 diabetes mellitus
Source: PeerJ. 2021 Apr 1;9:e11128. doi: 10.7717/peerj.11128 (PMC8019531; doi:10.7717/peerj.11128)
Supplement: Table S4 [file peerj-09-11128-s007.docx]

Table s4. A summary of the pyrosequencing data.

|  | L0 (n=40) | L4 (n=40) |
| --- | --- | --- |
| reads | 99228.15±4120.11 | 102025.23±3409.89 |
| OTUs | 364.30±31.54 | 228.13±15.60 |
| Phylum | 33 | 23 |
| Class | 86 | 56 |
| Order | 166 | 105 |
| Family | 257 | 167 |
| Genus | 444 | 308 |

OUT, operational taxonomic unit; L0, before treatment liraglutide; L4 after treatment liraglutide for 4 months.
