# Supplementary material for: Liraglutide-induced structural modulation of the gut microbiota in patients with type 2 diabetes mellitus
Source: PeerJ. 2021 Apr 1;9:e11128. doi: 10.7717/peerj.11128 (PMC8019531; doi:10.7717/peerj.11128)
Supplement: Table S6 [file peerj-09-11128-s009.docx]

Table s6. Clinical characteristics for the 40 individuals with T2DM enrolled in this study.

| Clinical features | Total |
| --- | --- |
| Age |  |
| <50 | 23 |
| ≥50 | 17 |
| Sex |  |
| Male | 31 |
| Female | 9 |
| BMI (kg/m^2^) |  |
| 25-30 | 22 |
| >30 | 18 |
| Family history of diabetes |  |
| Yes | 13 |
| No | 27 |
| Diabetes duration (years) |  |
| Short-duration diabetes (＜5 years) | 17 |
| Medium-duration diabetes (5-10 years) | 12 |
| Long-duration diabetes (≥10 years) | 11 |
| Family history of cardiovascular disease |  |
| Yes | 9 |
| No | 31 |
| Smoking |  |
| Yes | 19 |
| No | 21 |
| Alcoholism |  |
| Yes | 11 |
| No | 29 |
| Diabetic nephropathy |  |
| Yes | 9 |
| No | 31 |
| Diabetic retinopathy |  |
| No retinopathy | 26 |
| Mild non-proliferative diabetic retinopathy (NPDR) | 3 |
| Moderate NPDR | 9 |
| Severe NPDR | 2 |
| Diabetic peripheral neuropathy |  |
| Yes | 23 |
| No | 17 |

BMI, body mass index.
